# Tool Frame Calibration for Robot-Assisted Ultrasonic Testing

**DOI:** 10.3390/s23218820

**Published:** 2023-10-30

**Authors:** Hanming Zhang, Jingpin Wang, Canzhi Guo

**Affiliations:** 1 School of Mechanical and Materials Engineering, North China University of Technology, Beijing 100041, China; 2 School of Mechanical Engineering, Jiangsu University, Zhenjiang 212013, China; guocanzhi@ujs.edu.cn

**Keywords:** ultrasonic testing, robot-assisted, calibration, tool frame

## Abstract

Tool frame calibration has been widely used in robot-assisted printing, welding, and grinding, but it is not suitable for ultrasonic testing because the robot is submerged in water. The purpose of this paper is to present a tool frame calibration method, which is suitable for improving the precision of ultrasonic testing. In uniform mediums, sound travels along a straight line like ray. A reflector is fixed in water to reflect ultrasound, which makes it possible to measure distances between incidence points on a reflector and tool center point (TCP) on an ultrasound transducer. In addition, the positions and poses of the end flange are recorded through a robot controller. Finally, an optimization method is applied to calculate the position and pose errors of the tool frame relative to the end flange according to such records. The presented method was implemented in an ultrasonic testing system. We selected 100 incidence points on the reflector to calculate the assembly errors of the transducer. The pulse amplitude rose obviously after calibration, which verifies that this is an effective method. Considering that ultrasonic transducers can be used as a measuring tool, this paper proposes a tool frame calibration method for ultrasonic testing robots without introducing other measuring devices, which draws the conclusion that tool frame can be calibrated through ultrasound.

## 1. Introduction

Ultrasonic testing (UT) is used to detect defects in materials based on the principle of ultrasonic propagation. In the early days, UT was mainly used to ensure product quality. Now, it has been improved for use as an evaluation approach to predict potential risks [1]. The part under test is placed in water and a multi-axial manipulator grasping transducer moves over the part to scan the surface. Frame-type scan machines are widely used as manipulators. However, there are some limitations to such machines when scanning complex structures [2]. Then, general industrial robots were introduced [3]. These are more flexible to achieve complex motions compared to frame-type scan machines.

It is necessary to teach robots paths before running. There are two kinds of teaching methods, i.e., on-line teaching and off-line teaching. Off-line teaching is operated in simulation software, which is more flexible and efficient compared to on-line teaching. However, nominal coordinates inputted in the software may deviate from actual situations due to manufacturing error, assembly error, and so on. These errors cause actual paths to deviate from simulated paths.

Tool assembly error is a cause of path deviation. Some tool frame calibrations have been designed to reduce deviations. For example, Xiaozhi et al. presented a matrix-solving hand-eye calibration method using a laser scanner as a measuring tool to enhance the robotic machining accuracy of complex free form surfaces [4]. Guo et al. calibrated kinematic parameters for an industrial robot using a laser displacement sensor [5]. Lin et al. proposed an enhanced automatic TCP calibration method based on a laser displacement sensor, achieving the purpose of being simple, economical, and time-saving [6]. Similarly, assembly errors in ultrasonic transducers will influence testing precision. It is necessary to design a method to calibrate tool frames for robot-assisted ultrasonic testing.

Many measuring devices with different complexity and cost have been applied in robot calibration. In the early days, some appropriative measuring devices were designed. For example, Renders et al. designed an experimental setup using one magnetostrictive sensor to measure longitudinal displacement and two magnetic transducers to measure transversal displacement [7]. Driels et al. designed a wire potentiometer to measure robot partial pose, which could be used to calibrate PUMA 560 robots [8]. With the development of sensor technology, some universal measuring devices with high precision were applied, including the displacement sensor [9], complementary metal oxide semiconductor (CMOS) camera [10], laser tracker [11], laser interferometer [12], coordinate measuring machine (CMM) [13], etc. On the other hand, some optimization methods for solving non-linear problems have been used for robot calibration. Linear least-squares algorithm was employed for parameter identification to calibrate selective compliance assembly robot arm (SCARA) and a tree-typed modular robot [14]. Omodei et al. used nonlinear optimization, iterative linearization, and extended Kalman filter to identify kinematic parameters of SCARA [15]. Peng et al. used minimal linear combinations to analyze end-effector error and then used linear forecast real-time error compensation to decrease the error [16].

In this research, an ultrasonic transducer grasped by a robot was used as measuring device. A kinematic model was built based on the D–H model. The distance between the transducer and reflector was measured according to time-of-flight (TOF). In error calculation, a constrained non-linear optimization problem, was translated into an unconstrained linear optimization problem. Finally, the assembly errors were identified after several iterations. The experimental results demonstrate that it was feasible to measure and calibrate this tool frame using an ultrasonic robot. The presented method makes it easier to calibrate tool frames for robot-assisted ultrasonic testing system.

## 2. System Construction

**Figure 1:**
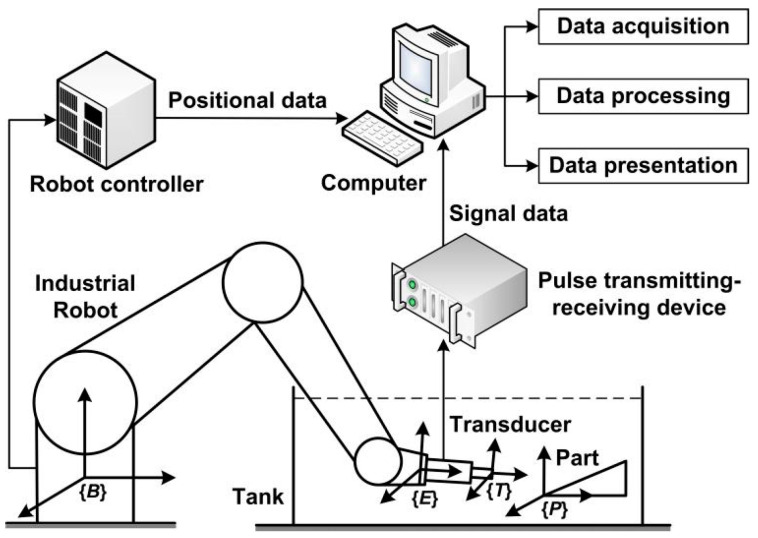
Configuration of robot-assisted ultrasonic testing system.

In 2002, Hongwei et al. designed an ultrasonic testing system using a frame-type scan machine as manipulator [17]. It was capable of scanning simple and continuous geometry, but useless for complex curved geometry. A six-degree-of-freedom (DOF)-articulated robot can make up for such shortcomings [18]. The construction of robot-assisted ultrasonic testing system is shown in Figure 1. It consists of three modules: the motion module, the testing module, and the auxiliary module. In the motion module, the industrial robot grasps the transducer scanning over the part. A robot controller is used for motion control and servo drive. The testing module consists of a pulse transmitting–receiving device and transducer. The auxiliary module includes a tank and some other support structures. Positional and signal data are fed into computer for data acquisition, processing, and presentation.

The robot is fixed on the ground and the part under test is placed in water. The transducer is installed on the end-effector of the robot. Frames are built on components, including the robot base frame {*B*}, end-effector frame {*E*}, tool frame {*T*}, and part frame {*P*}, to describe spatial relationships. All points on the parts are described relative to {*B*} for consistency. The tool frame calibration identifies errors between actual values and nominal values of the tool frame, and errors are due to assembly. Nominal values need to be adjusted during the off-line teaching.

## 3. Optimization-Based Calibration Method

The kinematic model describes the motion of objects without considering mass and forces. Denavit and Hartenberg presented the D–H model using four parameters associated with a particular convention to describe the position and pose of spatial links. Though many models were developed hereafter, such as the modified D–H (MDH) model, zero reference model, S model, and CPC model, the D–H model remains a popular approach. In this research, the D–H model is used to implement calibration. The values of the tool frame are calculated through an optimization algorithm. 

### 3.1. Robot Kinematic Model

**Figure 2:**
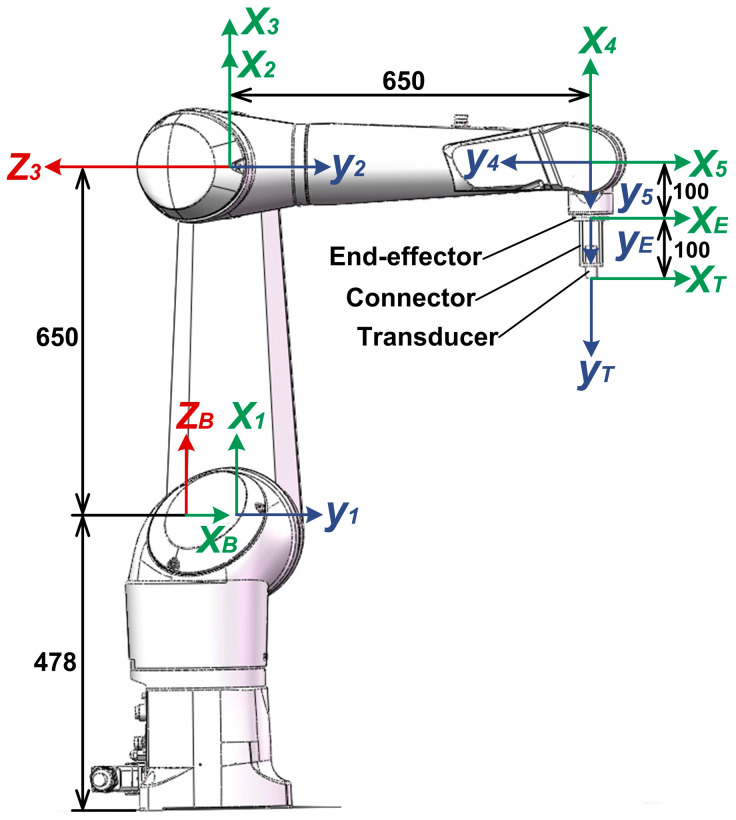
Structure and frames of Staubli-TX90XL industrial robot.

A Staubli-TX90XL industrial robot was used in this research and its structure is shown in Figure 2. The ultrasonic transducer was installed on end-effector of the robot. The frames were built on the base, links, end-effector, and transducer in line with the convention of the DH model.

The spatial relationship of two adjacent frames is described by homogeneous transformation matrix $T_{i-1}^{i}$:

(1) $$\begin{array}{l} T_{i-1}^{i}=Rot\left(Z,\theta_{i}\right)Trans\left(a_{i},0,d_{i}\right)Rot\left(X,\alpha_{i}\right)\\ \;=\left[\begin{array}{cccc} cos\theta_{i} & -sin\theta_{i}cos\alpha_{i} & sin\theta_{i}sin\alpha_{i} & a_{i}cos\theta_{i}\\ sin\theta_{i} & cos\theta_{i}cos\alpha_{i} & -cos\theta_{i}sin\alpha_{i} & a_{i}sin\theta_{i}\\ 0 & sin\alpha_{i} & cos\alpha_{i} & d_{i}\\ 0 & 0 & 0 & 1 \end{array}\right] \end{array}$$

where *θ_i_*, *ɑ_i_*, *d_i_* and *α_i_* in Equation (1) are DH parameters: *θ_i_* is the joint angle from the *X_i−_*_1_ axis to the *X_i_* axis about the *Z_i−_*_1_ axis; *ɑ_i_* is the offset distance from *the Z_i−_*_1_ axis to *the Z_i_* axis along *the X_i_* axis; *d_i_* is the joint distance from *the X_i−_*_1_ axis to *the X_i_* axis along *xZ_i−_*_1_ axis; *α_i_* is the offset angle from *the Z_i−_*_1_ axis to *the Z_i_* axis about *the X_i_* axis. The values of the D–H parameters are listed in Table 1.

Bringing above values into Equation (1), the spatial relationships of two adjacent links were determined as shown in Equation (2), where *c_i_* and *s_i_* are a simplification of *cosθ_i_* and *sinθ_i_.*

(2) $$\begin{array}{c} T_{0}^{1}=\left[\begin{array}{cccc} c_{1} & 0 & -s_{1} & a_{1}c_{1}\\ s_{1} & 0 & c_{1} & a_{1}s_{1}\\ 0 & -1 & 0 & d_{1}\\ 0 & 0 & 0 & 1 \end{array}\right]\;T_{1}^{2}=\left[\begin{array}{cccc} c_{2} & {-s}_{2} & 0 & a_{2}c_{2}\;\\ s_{2} & {\;c}_{2} & 0 & a_{2}s_{2}\;\\ 0 & \;0 & 1 & d_{2}\\ 0 & \;0 & 0 & 1 \end{array}\right]\\ T_{2}^{3}=\left[\begin{array}{cccc} c_{3} & 0 & s_{3} & \;0\;\\ s_{3} & 0 & -c_{3} & \;0\;\\ 0 & 1 & \;0 & \;0\;\\ 0 & 0 & \;0 & 1 \end{array}\right]\;T_{3}^{4}=\left[\begin{array}{cccc} c_{4} & \;0 & \;s_{4} & 0\;\\ s_{4} & \;0 & -c_{4} & 0\;\\ 0 & \;1 & 0 & {\;d}_{4}\;\\ 0 & \;0 & 0 & 1 \end{array}\right]\\ T_{4}^{5}=\left[\begin{array}{cccc} c_{5} & 0 & s_{5} & \;0\;\\ s_{5} & 0 & \;-c_{5} & 0\;\\ 0 & 1 & \;0 & 0\;\\ 0 & 0 & \;0 & 1\; \end{array}\right]\;T_{5}^{6}=\left[\begin{array}{cccc} \;c_{6} & {-s}_{6} & 0 & 0\;\\ \;s_{6} & {\;c}_{6} & 0 & \;0\;\\ 0 & 0 & 1 & {\;d}_{6}\;\\ 0 & 0 & 0 & 1 \end{array}\right] \end{array}$$

Furthermore, the spatial relationships of two arbitrary links were calculated through matrix multiplication. For example, the relationship between frames {*E*} and {*B*} in Figure 2 is expressed as

(3) $$T_{B}^{E}=T_{B}^{1}T_{1}^{2}T_{2}^{3}T_{3}^{4}T_{4}^{5}T_{5}^{E}=\left[\begin{array}{cccc} n_{x}^{E} & o_{x}^{E} & a_{x}^{E} & p_{x}^{E}\\ n_{y}^{E} & o_{y}^{E} & a_{y}^{E} & p_{y}^{E}\\ n_{z}^{E} & o_{z}^{E} & a_{z}^{E} & p_{z}^{E}\\ 0 & 0 & 0 & 1 \end{array}\right]$$

Equation (3) is simplified as

(4) $$\left[\begin{array}{ccc} n_{x}^{E} & o_{x}^{E} & a_{x}^{E}\\ n_{y}^{E} & o_{y}^{E} & a_{y}^{E}\\ n_{z}^{E} & o_{z}^{E} & a_{z}^{E} \end{array}\right]=R_{B}^{E},\;\left[\begin{array}{c} p_{x}^{E}\\ p_{y}^{E}\\ p_{y}^{E} \end{array}\right]=P_{B}^{E}\;\Rightarrow \;T_{B}^{E}=\left[\begin{array}{cc} R_{B}^{E} & P_{B}^{E}\\ 0 & 1 \end{array}\right]$$

where $R_{B}^{E}$ and $P_{B}^{E}$ describe the pose and position of {*E*} relative to {*B*}. Similarly, the relationship between {*T*} and {*B*} is expressed as

(5) $$T_{B}^{T}=T_{B}^{1}T_{1}^{2}T_{2}^{3}T_{3}^{4}T_{4}^{5}T_{5}^{E}T_{E}^{T}=\left[\begin{array}{cc} R_{B}^{T} & P_{B}^{T}\\ 0 & 1 \end{array}\right]$$

where $R_{B}^{T}$ and $P_{B}^{T}$ describe the pose and position of {*T*} relative to {*B*}; $T_{E}^{T}$ describes spatial relationship between {*T*} and {*E*}. The tool frame calibration in this research was to correct values in $T_{E}^{T}$.

### 3.2. Error Calibration

Laser trackers, CMM, and other measuring devices have been widely used to calibrate the tool frame in robotic systems. In this research, an ultrasonic transducer was introduced to calibrate the tool frame, which is uncommon in previous reports.

**Figure 3:**
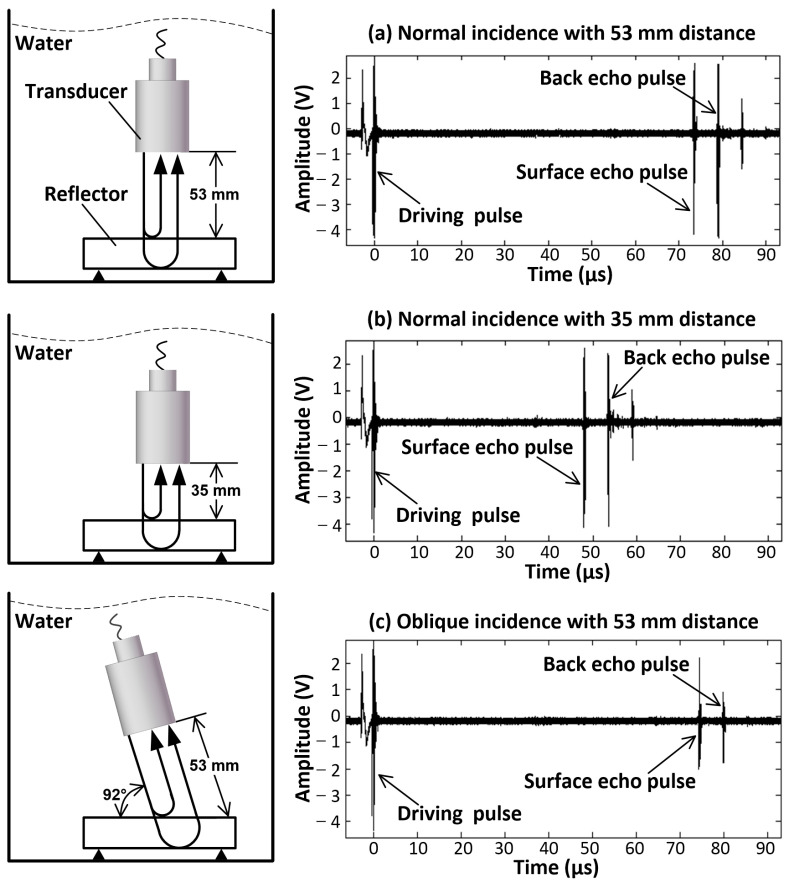
Waveform of ultrasonic with different distance and angle.

Ultrasonic transducers are sound–electricity converting components based on the piezoelectric effect in semiconductor elements. In the transmitting state, the pulse transmitting–receiving device transmits a narrow-pulse to drive the transducers. Then, the device turns into the receiving state and a series of echoes is received and converted into electrical signal. The driving pulse and subsequent echoes received with different incidence distances and angles are shown in Figure 3. Comparing Figure 3a,b, the TOF between the driving pulse and surface echo pulse changes linearly with the distance between transducer and reflector; comparing Figure 3a,c, the amplitude of the echo pulse decreases when the incident beam deflects a certain angle from the normal direction. Consequently, the distance between the transducer and the reflector can be measured based on the principle of ultrasonic propagation when it is conducted in homogeneous mediums like water.

**Figure 4:**
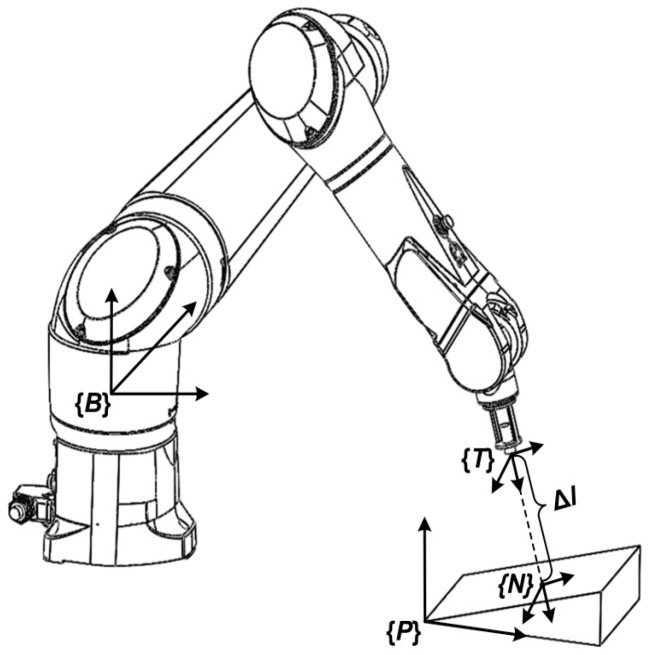
Measuring distance between transducer and reflector.

In Figure 4, there is a fixed spatial relationship between the robot and the reflector. The ultrasonic beam transmits Δ*l* along *Z* axis of {*T*} and intersects at point *N* on the reflector. A frame {*N*} is fixed at point *N* to describe the position and pose of *N* in space. It can be inferred from Equation (5) and coordinate conversion that the position and pose of *N* relative to {*B*} is

(6) $$T_{B}^{N}=T_{B}^{E}T_{E}^{T}\Delta L\;\Rightarrow \left[\begin{array}{cc} R_{B}^{N} & P_{B}^{N}\\ 0 & 1 \end{array}\right]=\left[\begin{array}{cc} R_{B}^{E} & P_{B}^{E}\\ 0 & 1 \end{array}\right]\left[\begin{array}{cc} R_{E}^{T} & P_{E}^{T}\\ 0 & 1 \end{array}\right]\Delta L$$

The elements of the matrixes in Equation (6) are

$$T_{B}^{N}=\left[\begin{array}{cccc} n_{x}^{N} & o_{x}^{N} & a_{x}^{N} & p_{x}^{N}\\ n_{y}^{N} & o_{y}^{N} & a_{y}^{N} & p_{y}^{N}\\ n_{z}^{N} & o_{z}^{N} & a_{z}^{N} & p_{z}^{N}\\ 0 & 0 & 0 & 1 \end{array}\right]\;T_{B}^{E}=\left[\begin{array}{cccc} n_{x}^{E} & o_{x}^{E} & a_{x}^{E} & p_{x}^{E}\\ n_{y}^{E} & o_{y}^{E} & a_{y}^{E} & p_{y}^{E}\\ n_{z}^{E} & o_{z}^{E} & a_{z}^{E} & p_{z}^{E}\\ 0 & 0 & 0 & 1 \end{array}\right]\;T_{E}^{T}=\left[\begin{array}{cccc} n_{x}^{T} & o_{x}^{T} & a_{x}^{T} & p_{x}^{T}\\ n_{y}^{T} & o_{y}^{T} & a_{y}^{T} & p_{y}^{T}\\ n_{z}^{T} & o_{z}^{T} & a_{z}^{T} & p_{z}^{T}\\ 0 & 0 & 0 & 1 \end{array}\right]\;\Delta L=\left[\begin{array}{cccc} \;1 & 0 & 0 & 0\\ \;0 & 1 & 0 & 0\\ \;0 & 0 & 1 & \;\Delta l\\ \;0 & 0 & 0 & 1 \end{array}\right]$$

The elements above have following relationship:

(7) $$\left(\begin{array}{c} p_{x}^{N}\\ p_{y}^{N}\\ p_{z}^{N} \end{array}\right)=\left(\begin{array}{c} p_{x}^{E}\\ p_{y}^{E}\\ p_{z}^{E} \end{array}\right)+\left(\begin{array}{ccc} n_{x}^{E} & o_{x}^{E} & a_{x}^{E}\\ n_{y}^{E} & o_{y}^{E} & a_{y}^{E}\\ n_{z}^{E} & o_{z}^{E} & a_{z}^{E} \end{array}\right)\left(\begin{array}{c} \Delta la_{x}^{T}+p_{x}^{T}\\ \Delta la_{y}^{T}+p_{y}^{T}\\ \Delta la_{z}^{T}+p_{z}^{T} \end{array}\right)$$

Among which, ($n_{x}^{E}$, $n_{y}^{E}$, $n_{z}^{E}$), ($o_{x}^{E}$, $o_{y}^{E}$, $o_{z}^{E}$), ($a_{x}^{E}$, $a_{y}^{E}$, $a_{z}^{E}$), and ($p_{x}^{E}$, $p_{y}^{E}$, $p_{z}^{E}$) are calculated according to the position feedback from the robot controller and Δ*l* is measured by the transducer. ($a_{x}^{T}$, $a_{y}^{T}$, $a_{z}^{T}$) is a unit vector that satisfies a constrain:

(8) $${\left(a_{x}^{T}\right)}^{2}+{\left(a_{y}^{T}\right)}^{2}+{\left(a_{z}^{T}\right)}^{2}=1$$

All incidence points on the reflector, such as *N*, are in line with a plane equation. Assuming that, there are three points on the reflector, noted as *A* (*x_A_*, *y_A_*, *z_A_*), *B* (*x_B_, y_B_, z_B_*), and *C (x_C_, y_C_*, *z_C_)*. The normal vector of the reflector is described as

(9) $$\begin{array}{l} n=\left(n_{x},n_{y},n_{z}\right)\\ \;=\left[\left(y_{B}-y_{A}\right)\left(z_{C}-z_{A}\right)-\left(z_{B}-z_{A}\right)\left(y_{C}-y_{A}\right)\right]i\\ \;+\left[\left(z_{B}-z_{A}\right)\left(x_{C}-x_{A}\right)-\left(x_{B}-x_{A}\right)\left(z_{C}-z_{A}\right)\right]j\\ \;+\left[\left(x_{B}-x_{A}\right)\left(y_{C}-y_{A}\right)-\left(y_{B}-y_{A}\right)\left(x_{C}-x_{A}\right)\right]k \end{array}$$

The plane equation is determined through the coordinates of *A*, *B*, and *C*, which are described as

(10) $$n_{x}\left(x-x_{A}\right)+n_{y}(y-y_{A})+n_{z}(z-z_{A})=0$$

Point ***N*** ($p_{x}^{N}$, $p_{y}^{N}$, $p_{z}^{N}$) shown in Figure 4 is in line with Equation (10):

(11) $$n_{x}\left(p_{x}^{N}-x_{A}\right)+n_{y}(p_{y}^{N}-y_{A})+n_{z}(p_{z}^{N}-z_{A})=0$$

The values of ***t*** ($p_{x}^{T}$, $p_{y}^{T}$, $p_{z}^{T}$, $a_{x}^{T}$, $a_{y}^{T}$, $a_{z}^{T}$) are unknown when taking Equation (7) into Equation (11). A measure and calculation process was designed to acquire the values.

The measuring process is shown in Figure 4. *m* incidence points were selected on the reflector. The transducer grasped by the robot was moved to the selected points with different poses. The spatial data of {*E*} relative to {*B*} were recorded in each point, and then the data were taken into Equation (11) and an optimization problem was established as

(12) $$Objective\;function:f\left(t\right)={\sum_{i=1}^{m}\left[n_{x}\left(p_{x}^{i}-x_{A}\right)+n_{y}(p_{y}^{i}-y_{A})+n_{z}(p_{z}^{i}-z_{A})\right]}^{2}$$

(13) $$Constrain\;function:c\left(t\right)={\left(a_{x}^{T}\right)}^{2}+{\left(a_{y}^{T}\right)}^{2}+{\left(a_{z}^{T}\right)}^{2}-1=0$$

Equations (12) and (13) constitute a non-linear optimization problem, which is difficult to solve directly. They were transformed into a linear optimization problem without constraint, which can be solved according to essential and sufficient conditions.

According to first-order essential condition, the objective and constrain function were rewritten together making up a Lagrange function:

(14) $$L\left(t,\lambda \right)=f\left(t\right)+\lambda \cdot c\left(t\right)$$

among which *λ* is noted as a Lagrange multiplier. Assuming that t* is an optimal solution, *f*(***t***) and *c*(***t***) are continuous and differentiable in the neighbourhood of t*, then there is a λ* satisfying:

(15) $$\nabla L\left(t^{*},\lambda^{*}\right)=\nabla f(t^{*})+\lambda^{*}\cdot \nabla c(t^{*})=0$$

Equation (15) transforms a non-linear optimization problem with equality constraint into a linear optimization problem that is solved by iteration. While t* acquired from iterations could be an extremal point, it needed to be further confirmed through second-order sufficient condition. The Hesse matrix relative to the Lagrange function was used. If it was a positive definite matrix when taking t* into Hesse matrix, then t* was confirmed as an extremal point. Consequently, t* was the corrected coordinates of {*T*} relative to {*E*}, which are the calibration results.

## 4. Experiment Results

**Figure 5:**
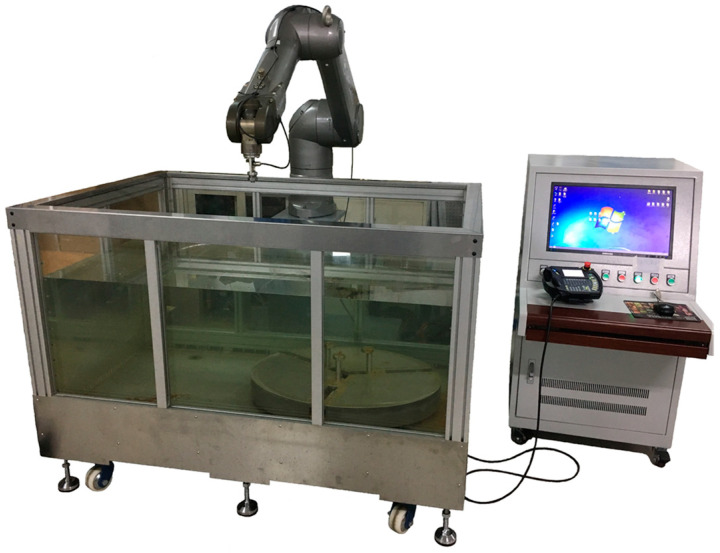
Experimental platform based on Staubli robot.

An experimental platform in line with Section 2 was established, as shown in Figure 5, to verify the proposed calibration. The industrial robot used in the experiment was produced by Staubli International AG (Pfäffikon, Switzerland), model TX90XL. The pulse transmitting–receiving device is an ultrasonic pulser/receiver produced by Olympus Corporation (Tokyo, Japan), model 5077PR. An ultrasonic transducer with a frequency of 25 MHz and an element size of 6 mm was selected. The model of the transducer is V324-SU-F5.25IN, which was also produced by Olympus Corporation.

**Figure 6:**
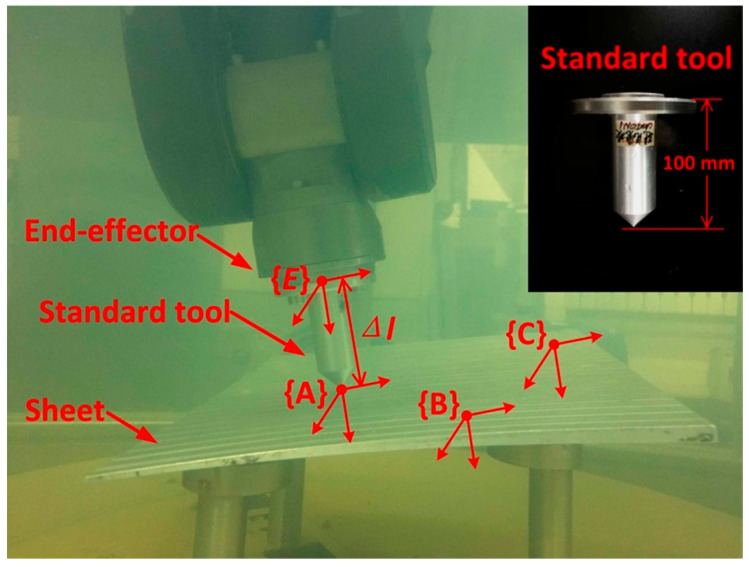
Contact measuring through standard tool to acquire plane equation.

It was demonstrated in Section 3 that all the incidence points on the reflector in line with a plane equation and the equation can be determined through three points on the reflector. Contact measuring was implemented, as shown in Figure 6, to acquire the coordinates of the three points. A standard tool was installed on the end-effector and a board (20 mm × 30 mm) was fixed in water.

The specific measuring steps were as follows:

Step 1 The robot is operated by making the standard tool contact the board with an arbitrary pose;
Step 2 The position coordinates of the contact point (such as *A*) relative to {*B*} are calculated through equation (6);
Step 3 Step 1 and 2 are repeated three times to acquire the position coordinates of point A, B, and C.
Step 4 The plane equation is calculated according to Equation (10).

Measuring steps 1–4 are repeated three times to reduce the influence of deformation when contacting the board. In addition, the standard tool can be replaced with a laser sensor to avoid deformation, but the measuring steps need to be implemented before pouring water into the tank. The coordinates of points are shown in Table 2. The plane equation calculated according to the points is

(16) −0.0088x−0.13y+0.99z+864.81=0

**Figure 7:**
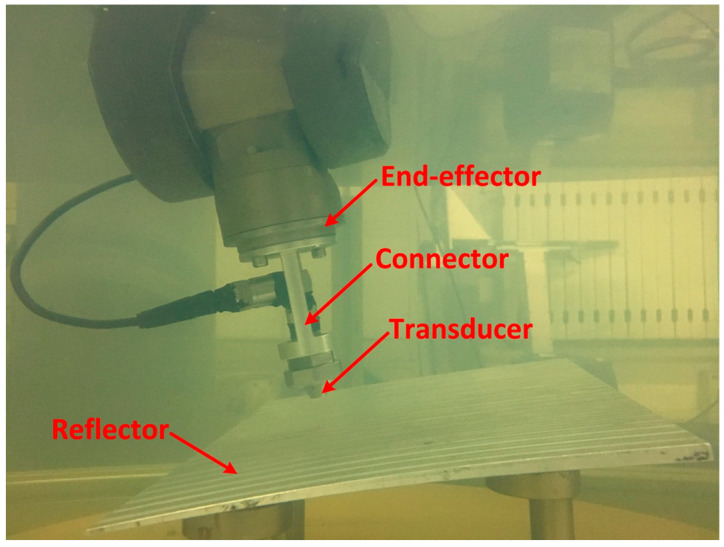
Non-contact measuring through ultrasonic transducer to acquire distance.

Then, the standard tool was replaced with an ultrasonic transducer and the board was used as a reflector as shown in Figure 7. The robot was operated by moving over the reflector with a different pose. During the operation 100 points and corresponding TOF were recorded.

**Figure 8:**
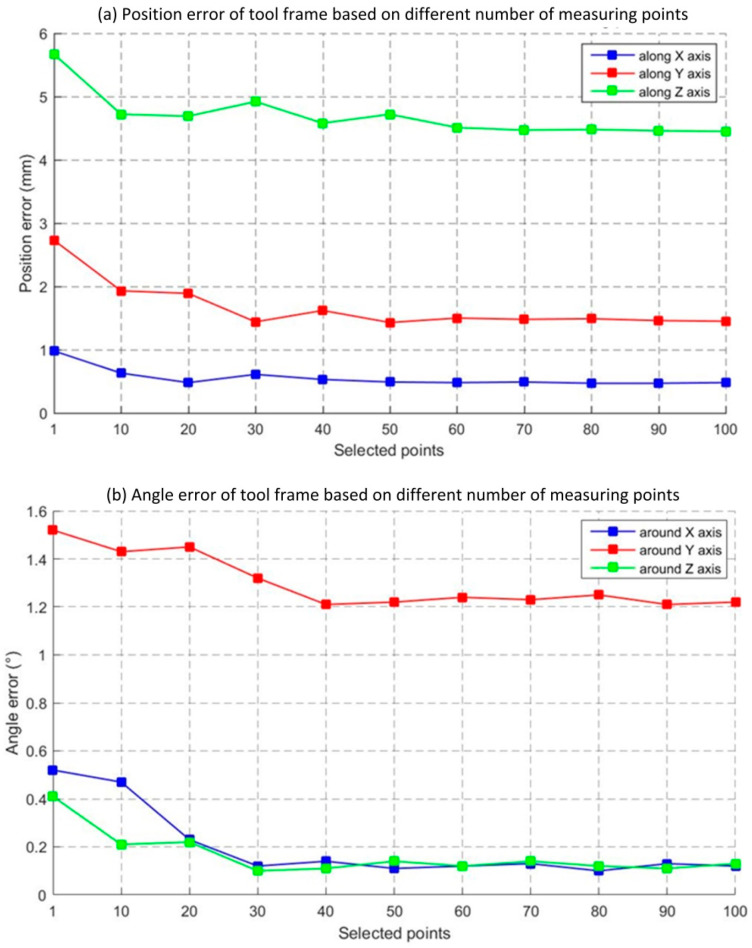
Position and angle errors of tool frame with different number of measuring points.

Several points were selected from 100 incidence points to calculate the coordinates of the tool frame based on the optimization algorithm presented in Section 3. The number of selected points was increased by ten each time from 1 to 100. The position errors along each axis and the angle errors around each axis between the calculated values and nominal values are shown in Figure 8a,b. The trend in the curves in Figure 8 demonstrate that the position and angle error decreased with the number of selected points. Consequently, fewer points can not obtain stable results and the calculated results’ trend become stable when the selected points are greater than 70. The calibration results are shown in Table 3.

**Figure 9:**
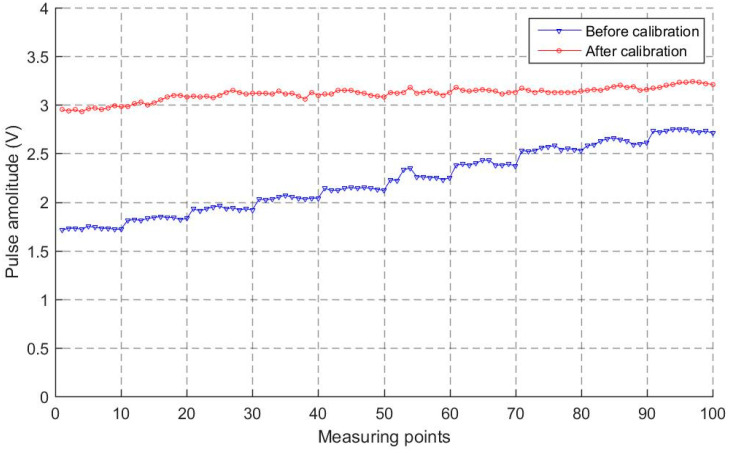
Comparison of pulse amplitude before and after calibration.

If there is a slight offset between the beam and the normal direction of the reflector, pulse amplitude will descend as shown in Figure 3c. This principle can be used to verify the effectiveness of the calibration method. The pulse amplitudes of the 100 incidence points before and after calibration are shown in Figure 9. The pulse amplitude of each measuring point after calibration is higher than that before calibration, which demonstrates that this is an effective calibration method.

## 5. Conclusions

Assembly errors in the transducer installed on a robot will influence ultrasonic testing precision. In this work, a tool frame calibration method based on ultrasonic principles is presented. The tool frame can be calibrated using a piece of board, which is economical and easily operated. Four conclusions are drawn as follows:

(1) A robot-assisted system which is more suitable to test complex curved parts compared with frame-type scan machines is proposed.
(2) The DH model is used to describe the statical relationship of each component. The tool frame calibration is modelled and translated into a mathematical problem to calculate the values in $T_{E}^{T}$.
(3) The calculation is described as a constrained non-linear optimization problem, then it is transformed into a linear optimization problem that can be solved by iteration.
(4) In this experiment, we find that the incidence points should be scattered on the reflector; the propagation distance should be different on each incidence point; and the transducer pose should be distributed in different angles.

The ultrasonic transducer can only measure distance in a single direction. Assembly errors are calculated by iteration. Though the calibration accuracy of this method is lower than that of universal measuring devices, such as laser trackers or coordinate measuring machines, it is suitable for underwater applications. Our future work will aim at improve the iteration accuracy.

## Figures and Tables

**Table 1:** DH Parameters of Staubli-TX90XL industrial robot.

Link	*θ_i_* [°]	*d_i_* [mm]	*a_i_* [mm]	*α_i_* [°]
1	*θ*_1_	0	50	−90
2	*θ*_2_−90°	50	650	0
3	*θ*_3_	0	0	90
4	*θ*_4_	−650	0	90
5	*θ*_5_−90°	0	0	90
6	*θ*_6_	100	0	0

**Table 2:** Coordinates of nine points.

*i*	*x_i_* (mm)	*y_i_* (mm)	*z_i_* (mm)
1	1035.06	366.72	−814.92
2	1035.04	519.10	−794.92
3	992.91	509.89	−796.71
4	992.96	341.27	−819.36
5	956.59	361.46	−817.28
6	956.56	536.33	−793.95
7	836.25	536.20	−795.29
8	844.11	371.18	−817.68
9	726.88	384.30	−816.16

**Table 3:** Tool frame calibration results.

Axis	Position Error (mm)	Angle Error (mm)
X	0.48	0.12
Y	1.48	1.22
Z	4.45	0.13

## Data Availability

The data are contained within the article.

## References

[1] Liu S., Liu F., Yang Y., Li L., Li Z. (2020). Nondestructive Evaluation 4.0: Ultrasonic Intelligent Nondestructive Testing and Evaluation for Composites. Res. Nondestruct. Eval..

[2] Mineo C., MacLeod C., Morozov M., Pierce S.G., Lardner T., Summan R., Powell J., McCubbin P., McCubbin C., Munro G. Fast ultrasonic phased array inspection of complex geometries delivered through robotic manipulators and high speed data acquisition instrumentation. Proceedings of the IEEE International Ultrasonics Symposium (IUS).

[3] Guo C., Xu C., Hao J., Xiao D., Yang W. (2019). Ultrasonic Non-Destructive Testing System of Semi-Enclosed Workpiece with Dual-Robot Testing System. Sensors.

[4] Feng X., Tian D., Wu H., Qian C., Zhu D. (2023). A matrix-solving hand-eye calibration method considering robot kinematic errors. J. Manuf. Process.

[5] Guo Y., Song B., Tang X.Q., Zhou X.D., Xie Y.L., Jin J. Calibration for kinematic parameters of industrial robot by a laser displacement sensor. Proceedings of the International Conference on Control, Automation, Robotics and Vision (ICARCV).

[6] Lin C.J., Wang H.C., Wang C.C. (2023). Automatic Calibration of Tool Center Point for Six Degree of Freedom Robot. Actuators.

[7] Renders J.M., Rossignol E., Becquet M., Hanus R. (1991). Kinematic calibration and geometrical parameter identification for robots. IEEE T. Robotic. Autom..

[8] Driels M.R., Swayze W.E. (1994). Automated partial pose measurement system for manipulator calibration experiments. IEEE Trans. Robot. Autom..

[9] Liu Y., Zhuang Z., Li Y. (2022). Closed-Loop Kinematic calibration of robots using a six-point measuring device. IEEE Trans. Instrum. Meas..

[10] Tang X., Zhou H., Xu T. (2023). A geometric errors identification method for the rotating axis of five-axis welding equipment. Int. J. Precis. Eng. Manuf..

[11] Xiao P., Ju H., Li Q., Meng J., Chen F. (2020). A new fixed axis-invariant based calibration approach to improve absolute positioning accuracy of manipulators. IEEE Access.

[12] Soichi I., Ryota U. (2022). A novel error mapping of bi-directional angular positioning deviation of rotary axes in a SCARA-type robot by “open-loop” tracking interferometer measurement. Precis. Eng..

[13] Gharaaty S., Shu T., Joubair A., Xie W., Bonev I.A. (2018). Online pose correction of an industrial robot using an optical coordinate measure machine system. Int. J. Adv. Robot. Syst..

[14] Chen I.-M., Yang G., Tan C.T., Yeo S.H. (2001). Local POE model for robot kinematic calibration. Mech. Mach. Theory.

[15] Omodei A., Legnani G., Adamini R. (2000). Three Methodologies for the Calibration of Industrial Manipulators: Experimental Results on a SCARA Robot. J. Robot. Syst..

[16] Chang P., Li C., Li T. (2011). Kinematic calibration and forecast error compensation of a 2-DOF planar parallel manipulator. Chin. J. Mech. Eng..

[17] Ma H.W., Zhang X.H., Wei J. (2002). Research on an ultrasonic NDT system for complex surface parts. J. Mater. Process. Technol..

[18] Aparicio Secanellas S., Gauna León I., Parrilla M., Acebes M., Ibáñez A., de Matías Jiménez H., Martínez-Graullera Ó., Álvarez de Pablos A., González Hernández M., Anaya Velayos J.J. (2023). Methodology for the Generation of High-Quality Ultrasonic Images of Complex Geometry Pieces Using Industrial Robots. Sensors.

